# Diabetes and COVID-19; A Bidirectional Interplay

**DOI:** 10.3389/fendo.2022.780663

**Published:** 2022-02-17

**Authors:** Paraskevi Kazakou, Vaia Lambadiari, Ignatios Ikonomidis, Aikaterini Kountouri, Georgios Panagopoulos, Stavros Athanasopoulos, Eleni Korompoki, Ioannis Kalomenidis, Meletios A. Dimopoulos, Asimina Mitrakou

**Affiliations:** ^1^ Diabetes Centre, Department of Clinical Therapeutics, Alexandra Hospital, School of Medicine, National and Kapodistrian University of Athens, Athens, Greece; ^2^ Second Department of Internal Medicine, Attikon University Hospital, National and Kapodistrian University of Athens, Medical School, Athens, Greece; ^3^ Laboratory of Preventive Cardiology, Second Cardiology Department, Attikon University Hospital National and Kapodistrian University of Athens, Medical School, Athens, Greece; ^4^ Department of Clinical Therapeutics, Alexandra Hospital, School of Medicine, National and Kapodistrian University of Athens, Athens, Greece; ^5^ 1^st^ Department of Intensive Care, Evangelismos Hospital, National and Kapodistrian University of Athens, Medical School, Athens, Greece; ^6^ Unit of Hematology and Oncology, Department of Clinical Therapeutics, Alexandra Hospital, School of Medicine, National and Kapodistrian University of Athens, Athens, Greece

**Keywords:** diabetes, COVID-19, endothelial (dys)function, new onset diabetes, antidiabetic medication

## Abstract

There seems to be a bidirectional interplay between Diabetes mellitus (DM) and coronavirus disease 2019 (COVID-19). On the one hand, people with diabetes are at higher risk of fatal or critical care unit-treated COVID-19 as well as COVID-19 related health complications compared to individuals without diabetes. On the other hand, clinical data so far suggest that the severe acute respiratory syndrome coronavirus 2 (SARS-CoV-2) may result in metabolic dysregulation and in impaired glucose homeostasis. In addition, emerging data on new onset DM in previously infected with SARS-CoV-2 patients, reinforce the hypothesis of a direct effect of SARS-CoV-2 on glucose metabolism. Attempting to find the culprit, we currently know that the pancreas and the endothelium have been found to express Angiotensin-converting enzyme 2 (ACE2) receptors, the main binding site of the virus. To move from bench to bedside, understanding the effects of COVID-19 on metabolism and glucose homeostasis is crucial to prevent and manage complications related to COVID-19 and support recovering patients. In this article we review the potential underlying pathophysiological mechanisms between COVID-19 and glucose dysregulation as well as the effects of antidiabetic treatment in patients with diabetes and COVID-19.

## Introduction

Since the outbreak of COVID-19 pandemic, diabetes has been in the epicenter of identified risk factors. Epidemiological data have indicated that older adults with co-morbidities are at highest risk to develop severe COVID-19 and subsequent complications including death. Such comorbidities include DM, obesity, respiratory and cardiovascular disease - including hypertension and coronary artery disease (1–3). Based on early clinical data on 122.653 COVID-19 cases reported by the United States Centre for Disease Control and Prevention (CDC) one third of infected patients had at least one of the above conditions. Furthermore, individuals with an underlying condition account for 78% of intensive care unit (ICU) admissions and 94% of deaths. Among the comorbidities, DM was the most frequently reported (10,9% of cases) condition (4). In a recent summary report by the Chinese CDC of 72314 cases, people with DM had the second highest fatality rate (7,3%) after cardiovascular disease (10,5%) compared to a rate of 2,3% in the general population (5). Further studies from China, Europe and the USA have also shown that when people with DM get COVID-19 they are at higher risk to develop COVID-19 related complications, to require ICU admission or to die from the disease (6–8). Supporting these findings, a large UK cohort study facilitating a health analytics platform, confirmed the role of uncontrolled diabetes as a risk factor (adjusted HR 1.95) (9)

In a recent publication, we reported the early and late endocrine complications of COVID-19 (10). SARS-CoV-2 infection affects several pathophysiological pathways, during the disease course, eventually leading to late complications. From that review, it was already clear that more data are about to emerge which would unravel the pathophysiology that partners these two conditions and would evince the most effective among the available anti-diabetic arsenal.

And even though the underlying elaborate pathophysiological mechanisms between COVID-19 and diabetes are still under investigation, the fact that there is a bidirectional interplay involving stress-induced pathways commonly shared by COVID-19 and diabetes is more than clear.

Our aim is to report the rapidly accumulating evidence that sheds light not only into the causal relationship of diabetes and COVID-19 severity and vice versa, but also to the best available anti-diabetic treatments easing recovery from COVID-19 severity, details not previously reported in our own or similar reviews. To that end, we will present a narrative review of the most cited publications on COVID-19 and diabetes, putting the spot on endothelium and β-cell function and their potential to be the common ground in these two devastating diseases.

## How Does DM Worsen COVID-19 Clinical Outcomes?

Epidemiological studies have rendered COVID-19 severity attributable to several diabetes complications or comorbidities as risk factors. Coexisting microvascular and macrovascular complications of DM such as cardiovascular disease, heart failure, retinopathy and reduced renal function may be responsible for the increased poor COVID-19 outcomes and mortality following infection (11–13). Obesity, a prevalent diabetes comorbidity, affects the immune system by means of adipose tissue. Adipose tissue, all along with the liver and the stressed β-cells, induce synthesis of pro-inflammatory cytokines and adhesion molecules and activate many metabolic and hemodynamic pathways, growth factors, as well as intracellular signals that mediate generalized vasculopathy and systemic inflammation (14). Obesity highly correlates with T cell immune signatures that are unique for severe COVID-19, highlighting a potential contributing mechanism (15). However, if one tries to search for mechanisms attributable to diabetes per se, endothelium and chronic inflammation are the two main conceptual approaches, as we describe next.

Endothelium is considered a major player in multiple physiological processes, supporting homeostasis of healthy individuals. Particularly, it regulates the tone of vascular smooth muscle by the release of vasodilators and vasoconstrictors, controls vascular permeability and is an important mediator of inflammation and coagulation by regulating the adhesion of leucocytes and platelets (16–18). Endothelial glycocalyx is an endothelial surface layer which consists of proteoglycans and glycoproteins and prevents the direct contact of blood cells with the endothelial surface (19). DM is associated with chronic endothelial dysfunction and endothelial glycocalyx damage which increase leucocyte and circulating inflammatory cells adhesion and promote vascular permeability and coagulation (20–23). Hyperglycemia and insulin resistance have been suggested as possible mechanisms which lead to the impairment of the endothelial micromilieu in patients with DM. Acute and long-term hyperglycemia affects sorbitol, protein kinase C, and pentose phosphate pathways and this leads to increased oxidative stress and promotes endothelial cells apoptosis contributing to endothelial dysfunction. The decreased availability of NO is another mechanism through which hyperglycemia leads to endothelial dysfunction (24). Adequate glycaemic control has been associated with improved endothelial function. Our group recently reported a study in one hundred patients with type 2 DM (T2DM). We have shown that 12-months of intensified antidiabetic treatment improves arterial stiffness, endothelial glycocalyx and markers of oxidative stress. We found that high values of hemoglobin A1c (HbA1c) are positively corelated with the perfused boundary region (PBR), a marker of glycocalyx thickness. This finding indicates that hyperglycemia may lead to the impairment of glycocalyx integrity (25). Regarding the contribution of insulin resistance in endothelial dysfunction, a case-control study showed that Matsuda Index and insulin sensitivity index (ISI) are correlated with PBR. This implies that insulin resistance induces glycocalyx damage in first-degree relatives of diabetic patients (26). Chronic endothelial dysfunction predisposes to severe COVID-19 infection by inducing alterations to the glycocalyx and endothelial cells. This leads to increases in leucocyte adhesion and promotes a procoagulant and antifibrinolytic state. DM induced chronic endothelial dysfunction in combination with the direct damage of endothelial cells by SARS-CoV-2 results in endothelial and microcirculation impairment which in turn contribute to the pathogenesis of acute respiratory syndrome and multi-organ failure.

Diabetes, even at the stages of prediabetes, is characterized by a chronic inflammatory and prothrombotic state with multiple metabolic, vascular, immune, and hematological abnormalities which may affect adversely the response to infections (27–29). The glucotoxicity of diabetes coincides with impaired immunity; inhibition of lymphocyte proliferation, reduced natural killer cell activity, monocyte/macrophage and neutrophil dysfunction (28, 30, 31). In addition to the chronic endothelial effects of diabetes and the resulting susceptibility to severe COVID-19, hyperglycemia on admission and during hospitalization results in worse prognosis, severity, and mortality of COVID-19, irrespectively of prior diabetes status. Retrospective studies from China have demonstrated that elevated glucose levels on admission were an independent risk factor of progression to critical cases and in-hospital mortality (32, 33). Similarly, an observational study of more than 1,000 inpatients with COVID-19 at US hospitals showed that 40% of the subjects had diabetes or uncontrolled hyperglycemia on admission and inpatient mortality was fourfold greater for patients with DM. Another noteworthy finding of the same study was that mortality was sevenfold higher for those without pre-existing DM who developed hyperglycemia in hospital (34). A recent meta-analysis of 14502 patients confirmed these findings further and proved a nonlinear relationship between admission fasting blood glucose and severity: each increase in glucose level by one mmol/L, increased the risk of severity by 33% (35). Similar retrospective studies of hospitalized COVID-19 patients from China revealed that disease severity was correlated with active inflammatory markers as IL-6 and lactate dehydrogenase and that patients with DM had greater inflammatory response with higher IL-6, CRP, ESR and relative neutrophilia with lymphopenia as well as higher incidence of coagulopathy with higher D-dimer levels and longer prothrombin times (36, 37). Apart from the increased inflammatory response, DM induced hyperglycemia and increased glycolysis, promoted SARS-CoV-2 replication and increased the viral load. This increased viral replication is achieved through a mitochondrial ROS/hypoxia-inducible factor-1a dependent pathway, resulting in T cell dysfunction and epithelial cell death (38). Interestingly enough, in one retrospective study, hyperglycemia halted the beneficial effects of Tocilizumab which targets interleukin-6 receptor and reduces cytokine storm (39).

Such evidence coincides with earlier coronavirus outbreaks, where it was likely to see higher rates of hyperglycemia on admission, irrespective of pre-existing glycaemic status, disease severity or glucocorticoid use (40, 41). In a Chinese study, SARS-CoV, was shown to bind to ACE2 in pancreatic islet cells, damage them and cause acute hyperglycemia, even among people without DM. Approximately 50% of SARS patients without diabetes or steroid treatment developed DM during hospitalization, and after 3 years, 5% of the patients still had DM. These findings generated the hypothesis of chronic β-cell damage, as we present later on this review (41).

## How Does COVID-19 Worsen Endothelial Function and Glucose Homeostasis

SARS-CoV-2 can have an acute effect on the endothelium which as we have presented is already affected by diabetes as a chronic disease. SARS-CoV-2 affects the vascular system directly by targeting endothelial cells leading to severe endothelial derangement and inflammation. ACE2 receptors which are the main targets of SARS-CoV-2 are expressed in various human tissues including the endothelium, allowing the direct invasion of endothelial cells (42–44). In addition, the overproduction of pro-inflammatory cytokines during COVID-19 promotes endothelial dysfunction (45). Indeed, Chen et al., showed that patients with severe COVID-19 disease displayed increased levels of pro-inflammatory cytokines including soluble interleukin-2 receptor (IL-2R), interleukin-6 (IL-6), and tumor necrosis factor-α (TNF-a) (46). IL-6 promotes endothelial derangement and contribute to procoagulability (47, 48). Furthermore, inflammatory-mediated damage of glycocalyx by IL-6, or TNF-α increases vascular permeability inducing interstitial fluid shift and generalized oedema (49). Our group recently showed that SARS-CoV-2 may cause endothelial and vascular dysfunction linked to impaired cardiac performance four months following infection (50). To further support the role of pro-inflammatory cytokines in COVID-19 pathophysiology, another recent study showed that the inhibition of IL-6 by tocilizumab reverses lymphopenia in critically ill ventilated COVID-19 patients (51). Similarly, a randomized clinical trial, including 389 hospitalized patients with COVID-19 pneumonia, showed that treatment with tocilizumab reduces the risk for adverse clinical outcomes including mechanical ventilation or death (52). The infected endothelial cells release increased levels of proinflammatory cytokines which induce immune-mediated damage of lungs and other organs. This results in acute respiratory distress syndrome (ARDS) and multi-organ failure (53). Histological studies showed that endothelial cells damage by SARS-CoV-2 results in vasculitis and endothelitis in multiple organs (54–56). Post-mortem histological evidence of three patients were the first reports to highlight the role of endothelium in the pathogenesis of COVID-19 disease and provided evidence of endothelitis during infection. This suggests that the clinical presentation of COVID-19 might be worse in vulnerable patients with pre-existing endothelial dysfunction (54). Copin et al. showed that endothelial derangement induces vascular damage of small to medium-sized pulmonary arteries contributing to lung injury in severe COVID-19 (56).

On regards to glucose homeostasis, as we previously reported, severe COVID -19 with the accompanying cytokine storm and dysregulated counterbalancing hormonal responses, potentially contributes to insulin resistance and hyperglycemia (10, 57, 58). Similarly, viral respiratory infections have the potential to provoke a raised-interferon-γ-mediated insulin resistance in skeletal muscles (59). Experience from Severe Acute Respiratory Syndrome (SARS) and Middle East Respiratory Syndrome (MERS) suggests that inflammatory cells can affect the liver, impairing insulin-mediated glucose uptake, with hyperinsulinemia and hyperglycemia as a result (60, 61). Thus, in COVID-19 the viral inflammatory and immune responses can impair insulin sensitivity and dysregulate glucose metabolism, leading to a vicious cycle of hyperglycemia and inflammatory response that destroys tissue integrity and physiological function during the critical stages of infection. Of notable mention, drugs often used in the treatment of COVID-19, such as corticosteroids or antiviral agents, can aggravate hyperglycemia and result in lipodystrophy and insulin resistance (62, 63).

Acting together, glucotoxicity with its consequences in conjunction with the inflammatory cytokine storm of COVID-19 infection, and along with the increased oxidative stress, the immune dysfunction and the endothelial damage result in further metabolic complications, as increased risk of thromboembolism and multiorgan damage in individuals with DM (64–66).

## SARS-CoV-2 Infection and New-Onset DM

As it has already been mentioned, based on existing data, COVID-19 affects glucose homeostasis even in the absence of DM in one’s past medical history (10). Recent findings have suggested a direct effect of SARS-CoV-2 on glucose metabolism as new presentations of DM with diabetic ketoacidosis (DKA), hyperosmolarity and unusually high requirements of insulin to achieve glycaemic control have been reported (67–72). Based on our established experience on critical conditions, this amplified insulin resistance is unprecedented. Additionally, a COVID -19 infection has been found to precipitate acute hyperglycemic crises and to worsen prognosis for poorly controlled patients with DM (73). Therefore, in order to reduce the risk of metabolic complications, severe outcomes, and mortality during a Covid infection, it is crucial to achieve glycaemic control (67, 74).

A direct link on how SARS-CoV-2 might cause or worsen DM has not yet been established. Reports from Germany and Italy, including data from nationwide pediatric diabetes centers, have described higher frequency of DKA and severe DKA in children and youth with new-onset Type 1 DM (T1DM) during the pandemic, while the incidence of new-onset T1DM did not change (75–77). Higher frequency of DKA diagnosis might be attributed to postponed hospital visits and diagnosis of T1DM due to the overall overload in public healthcare services. Fear of contracting COVID-19 resulted in reduced visits to public health services and hindered access to routine clinical care (78). On the other hand, the postulation that COVID-19 could precipitate or accelerate the onset of T1DM is a fascinating-yet-plausible hypothesis. In favor of this notion, a Northwest London Study, described an 80% increase of new T1DM cases for children infected or exposed to SARS-CoV-2 among those that were assessed (79). Another case report presented a 19-year-old German male who was hospitalized with DKA and insulin-dependent diabetes in the absence of typical T1DM autoantibodies 5 to 7 weeks after asymptomatic SARS-CoV-2 infection (80). A plausible explanation is that SARS-CoV-2 infection might have damaged β-cells in the pancreas through direct cytolytic effect of the virus. Additionally, autoantibody negativity -although not rare- coexisting with a rare genetic variant, has raised suspicions favoring a new type of insulin-dependent diabetes. Caution is needed though, as the hyperglycemic state might be a pre-existing condition that had not yet been diagnosed, as it is implied by the HbA1c (16,8%) upon diagnosis. Two other reports however, demonstrated DKA with high HbA1c irrespective of the duration of DM (80, 81). As we previously reported briefly, the magnified psychological stress during the extended lockdowns, could in turn be an exacerbating factor in genetically susceptible individuals, and it is a factor not to be neglected (10). Currently, a global registry of COVID-19-related diabetes has been launched to shed light into the extent and characteristics of such new cases.

## SARS-CoV-2 Interaction With the Pancreas: The Role of ACE2

There seem to exist some possible underlying mechanisms which could explain the acute damage of the pancreatic islets by SARS-CoV-2 and the subsequent loss of insulin secretory capacity. An immune response *via* the release of chemokines and cytokines mediated by the virus, might also kill pancreatic cells and impair their ability to sense glucose and release insulin. Furthermore, the immune response of the virus may further impair the ability of the liver and muscles to identify alterations (67, 82).

Whether ACE2 is the main culprit for β-cell dysfunction has been a matter of scientific debate. A recent functional study has revealed that ACE2 is the main binding receptor for the SARS-CoV-2 spike glycoprotein, easing host cell entry (83). This receptor apart from the respiratory system is also expressed in key metabolic organs and tissues, including pancreatic β-cells, adipose tissue, the small intestine, and the kidneys (42). SARS-CoV-2 enters host cells after binding to ACE2 receptor, and both SARS-CoV-2 and ACE2 are internalized by endocytosis, so that surface ACE2 is then downregulated (84). This SARS-CoV-2 induced ACE2 under-expression and inflammatory injury seem to be extended in all cells with ACE2 activity, including pancreatic islets (85, 86). Elevated pancreatic enzymes that have been reported in COVID-19 are indicative of such damage (87–89). A study on organoids confirmed that the β-cell ACE2 expression results in SARS-CoV-2 tolerance which in turn leads to cytokine inflammation, β-cell apoptosis and eventually reduced insulin secretion (90). Another study that used human islet cell cultures and post-mortem examinations from SARS-CoV-2 infected individuals, confirmed the viral presence and replication in the islets, as well as a reduction of insulin-secretory granules and the impaired glucose dependent insulin secretion (91). Two more recent studies in autopsy samples showed a binary effect on β-cells, leading to either the death of β-cells or transdifferentiation of the surviving ones (92, 93). Preclinical studies also support the role of ACE2 in β-cell homeostasis; in high fat diet mice β-cell de-differentiation is characterized by reduction of ACE2; deletion of ACE2 in diabetic mice induces hyperglycemia, β-cell oxidative stress and decrease of insulin secretion (94, 95). Finally, ACE2 deletion in obese mice impairs β-cell proliferation, decreases β-cell mass and results in β-cell dysfunction (96, 97).

However, this might not describe the whole picture, as new evidence accumulates. What has already been established is the role of ACE2 and TMPRSS2 (transmembrane serine proteases 2) for the entry of SARS-Cov-2 in epithelial cells. Evidence from immunohistochemistry surveys suggests that they are expressed in microvasculature and ducts and only rarely -and at low levels- they are found in islet cells. (98, 99). Other investigators report an *in vitro* study where a preferential expression of ACE2 in β-cells, together with ACE2 upregulation, is observed during inflammation (100). Additional factors, namely HMBG1 (High-mobility group box 1) and NRP1 (neuropilin1), are crucial for SARS-CoV-2 infection and have been found to be also expressed in β-cells. HMGB1, a chromatin regulator, is critical in ACE2 expression, and is minimally expressed in COVID-19 patient’s islets compared to controls (101, 102). NRP1, which binds furin-cleaved substrates and potentiates SARS-CoV-2 infectivity has been detected in islets (101, 103). Since an intact microvasculature is a prerequisite for full islet functionality, the vascular damage facilitated by ACE2, seems to be the main culprit in β-cell dysfunction, potentially reinforced by additional factors.

If ACE2 is not the main receptor for SARS-CoV-2 to affect islets, and several others emerge among studies, a series of other underlying mechanisms render ACE2 crucial factor to understand the impairment of β-cell function. Acute hyperglycemia provokes ACE2 expression on cells and increases urinary ACE2 activity which may lead to an increase of viral load. Urinary ACE2 activity and protein levels are elevated in patients with T1DM as well as T2DM and urinary ACE2/Creatinine is positively correlated with fasting blood glucose and HbA1c (104, 105). Furthermore, chronic hyperglycemia in diabetes counteracts ACE2 expression perpetuating the inflammation and the severity of the infection (106). In patients with DM, SARS-CoV-2 through reducing ACE2 expression results in decreased degradation of angiotensin II and increased secretion of aldosterone and renal potassium loss (107). Hypokalemia can further blunt insulin secretion (63). Of special mention, dipeptyl peptidase 4 (DPP4), important in glucose homeostasis, is another functional coronavirus receptor, interacting with MERS-CoV (108–110). Although ACE2 is recognized as the main receptor, DPP4 could also bind to SARS-CoV-2, as suggested by viral modelling, but this is yet to be confirmed experimentally (111, 112).

## How May Antidiabetic Treatment Influence SARS-CoV-2 Infection in People With DM

Given all the valuable information from existing research it becomes apparent that glycaemic control can be crucial as a preventive measure of adverse COVID-19 related outcomes. However, when looking for the optimal therapeutic modalities both benefits and risks of glucose lowering agents should be taken into account.

Sodium-glucose cotransporter-2 inhibitors (SGLT2is) are used to treat T2DM by promoting urinary excretion of glucose and inducing osmotic diuresis. They are well tolerated and highly cardioprotective in the context of cardiac failure, and they also exhibit anti-inflammatory properties (113). Nevertheless, despite this being rare, patients with DM using SGLT2is are at increased risk for dehydration and euglycemic DKA (euDKA), during acute illness, especially in a setting of anorexia and vomiting (114, 115). The risk for euDKA seems even more enhanced during COVID-19. A case series of euDKA in patients with T2DM and COVID-19 while on SGLT2is has been reported recently (116). Specific precipitating factors for euDKA with the use of SGLT2is during COVID-19, apart from volume depletion due to vomiting and anorexia, may include a direct cytolytic effect of the virus on β-cells with consequent decreased endogenous insulin secretion and an increased inflammatory response with elevated IL-6 levels (117). Nonetheless, a hypothetical neutralizing effect of SGLT2is on COVID-19 infection has been suggested. This can occur by two distinct mechanisms. Firstly, SGLT2is might reduce viral load by increasing lactate concentrations and by decreasing intracellular pH. Secondly, they have known anti-inflammatory effects, especially on endothelial function (64). It is therefore hypothesized that SGLT2is could play a protective role in organ failure during COVID-19. However, in a recent double-blind placebo-controlled trial (DARE-19) of dapagliflozin in patients hospitalized with COVID-19 and having one or more cardiometabolic risk factors, the SGLT-2 did not confer a significant reduction in organ dysfunction or death, nor improved clinical recovery, regardless of its safety profile (118).

Thiazolidinediones are agonists of the peroxisome proliferator- activated receptor- γ (PPARγ), a nuclear receptor that regulates the transcription of various genes involved in glucose and lipid metabolism (119). They have been shown to improve insulin resistance and to possess anti-inflammatory and anti-atherosclerotic effects (120). However, they are not suitable for critically ill patients because they have been associated with fluid retention, oedema, and heart failure aggravation (121).

DPP4 inhibitors (DPP4is) are widely used in the treatment of T2DM as they have a negligible risk of hypoglycemia and are well tolerated. DPP4 is a transmembrane glycoprotein which is known to have a vital role in glucose homeostasis. It is expressed in the spleen, lung, liver, kidney, and immune cells. Furthermore, it can also be found in the circulation as soluble DPP4 (122). It is suggested that DPP4is could be of therapeutic benefit in mild or even in severe COVID-19 (123). The role of DPP-4 inhibition as a mitigator of inflammation and a potent anti-fibrotic agent, is supported by various experimental studies (122, 124). It is compelling to presume that such medication could reduce pro-inflammatory cytokine production during COVID-19. It has been shown after all that in ARDS they managed to reduce histological lung injury (125). DPP4 is also a known coreceptor for the MERS spike protein, and mice with higher DPP4 expression had more severe disease (109, 111). In addition, plasma levels of DPP4 in patients with MERS- CoV were significantly reduced, suggesting a protective role of DPP4 (126). Nevertheless, it is not known if SARS-CoV-2 uses DPP4 for cell entry and therefore it is unclear whether DPP4is interfere with this binding, and whether they reduce virulence (112). An interaction between DPP4 and SARS-CoV-2 spike protein has been shown, suggesting that DPP4 receptor binding domain could be a potential key for treating infection by SARS- CoV-2 (111, 127). In an *in vitro* study, treatment with DPP4is did not stop the entry of coronavirus into cells (109). In a retrospective case–control study from Italy, sitagliptin use during hospitalization was associated with reduced mortality and improved clinical outcomes in patients with T2DM and COVID-19 (128). Furthermore, another Italian case series found a significantly reduced mortality in eleven patients with DPP4is treatment while in another study DPP4i treatment was associated with worse outcomes in twenty-seven patients with T2DM compared to forty-nine treated with other glucose- lowering medications (129, 130). More detailed clinical trials are needed to fully evaluate the role of DPP4is in patients with T2DM and COVID-19.

Glucagon-like peptide-1 receptor agonists (GLP-1 RA) have been shown to reduce major cardiac events in patients with T2DM in clinical trials. They have also been found to exert anti-inflammatory actions in low-grade inflammation conditions such as atherosclerosis and non-alcoholic fatty liver disease (131, 132). In addition, they contribute to weight reduction in obesity, another state of chronic inflammation and compromised immune response (133). Of note, experimental studies have shown that they can reduce cytokine production and mediate pulmonary inflammation in mice (55). It could thus be hypothesized that they might be beneficial in COVID-19 infection since it is a state of hyperinflammation with worse outcomes in people with atherosclerosis, obesity or T2DM. Short-term studies of patients in the ICU have shown that such medication is safe and effective for blood glucose management (134). However, data is not sufficient to recommend these agents in critically ill patients with T2DM and COVID-19. There is always the risk of gastrointestinal side effects, such as nausea and vomiting and the risk of aspiration in such patients (106).

Metformin has already been found to have anti-inflammatory properties and to reduce inflammatory biomarkers, such as Il-6 and TNF-a in people with T2DM, as well as in an ARDS model (135–137). Since exacerbated inflammation due to cytokine storm has been recognized as a major key of bad prognosis in COVID-19, it could be hypothesized that metformin might have a beneficial role. Still though, the immunomodulatory actions of metformin in the context of COVID-19 remain unclear. Evidence for other possible positive implications of metformin in COVID-19 such as reduction in insulin resistance and inhibition of the virus entry through adenosine monophosphate-activated protein kinase (AMPK) activation and phosphorylation of ACE2 are also scant. There are few retrospective studies that have shown that metformin may have a positive effect on prognosis of hospitalized patients with T2DM and COVID-19 and especially on reduction of mortality, even though with a degree of heterogeneity (138, 139). In addition, a recent cohort study in the United Kingdom showed that metformin was not associated with the risk of COVID-19 or COVID-19-related mortality, indicating that continuation of metformin is safe in adults with diabetes (140). Randomized controlled trials (RCTs) are needed to draw more robust conclusions.

Sulfonylureas should be avoided in patients with DM and COVID-19 because of the risk of hypoglycemia, especially if oral intake is poor or with simultaneous use of chloroquine or hydroxychloroquine (66).

Insulin has been the first choice of treatment for glucose control in critically ill patients. In accordance, available evidence suggests that it is also the most appropriate glucose lowering therapy for hospitalized patients with DM and severe COVID-19 (64, 66, 106). Furthermore, it has been shown to have anti-inflammatory properties in acute illness, a fact that has also been observed in hospitalized patients with COVID-19 (141, 142). Recent studies have also shown that in many severe cases with new-onset or pre-existing DM with COVID-19, high doses of intravenous insulin infusion are needed to achieve glycaemic control (67–71) Fortunately, continuous glucose monitoring during hospitalization has contributed to achieve such glucose management with minimizing the risk of hypoglycemia. More than this, it has achieved improvement of glycaemic control in ICU hospitalized patients and offers the advantage of remote monitoring (143–146).

On regards to direct comparison of different medications, even though there are few data suggesting that insulin treatment in hospitalized diabetic patients with COVID-19 has worse clinical outcomes compared with other agents such as metformin, we should keep in mind that such an observation could reflect a more advanced stage of diabetes with accompanying comorbidities not allowing the use of other hypoglycemic medication (133, 147, 148). Similarly, a recent nationwide observational cohort study in England showed that T2DM patients prescribed metformin, SGLT2is and sulfonylureas were at a lower risk of COVID-19-related mortality than those who were not prescribed these drugs. The risk was higher in those prescribed insulin and DPP4is. However, since the differences in risk were small and due to confounding rather than direct drug effects, no evidence supports a change in prescription of glucose-lowering drugs in people with T2DM in the context of COVID-19 pandemic (149).

Hence, to make sense of available evidence, we suggest discontinuation of SGLT2is in patients who are severely affected by COVID-19 and who are at risk for dehydration (66, 106). Thiazolidinediones should be discontinued in hemodynamically unstable patients with cardiac or hepatic dysfunction. Their use in COVID-19 patients with DM is controversial and further studies are needed (64, 65). GLP-1 RA, until more detailed trials are available, should only be recommended in patients with mild or moderate COVID-19. Focusing on metformin, we should highlight that it should be stopped in cases of severe gastrointestinal symptoms. It should also be avoided in patients with severe COVID-19, hemodynamically unstable patients with hypoxia, hepatic, renal or heart failure because of the risk of lactic acidosis (66, 106). Current data support the use of DPP4is to be limited to mild or moderate COVID-19 cases in either inpatient or outpatient settings, but insulin should continue to remain the cornerstone of treatment in severely and critically ill patients (64, 66, 106).

## DM Post COVID-19

In addition to pancreatic and liver effects, damage of endocrine organs during the infection may contribute to the development of glucose and metabolic abnormalities in survivors of COVID-19 (10). People with ARDS and other critical conditions admitted to ICUs exhibit post-traumatic stress disorder with cognitive impairment, depression, impaired sensing of hunger and satiety, increased fat accumulation and increased risk of cardiovascular abnormalities (150). Furthermore, decreased exercise capacity and deranged lean muscle mass have been reported to diminish insulin sensitivity in survivors of ARDS and sepsis. Similarly, cachexia and muscle weakness are probable in severe and critical cases of COVID-19 (151, 152). Finally, rhabdomyolysis, which might contribute to long-term metabolic abnormalities, has been reported during the infection (153). In a study from China, subjects with SARS -CoV infection presented with metabolic disturbances including hyperlipidemia, cardiovascular abnormalities, abnormal glucose metabolism, such as hyperglycemia, hyperinsulinemia, insulin resistance, T1DM or T2DM twelve years after recovery (154). While the pandemic evolves over time, a cross-sectional study of 543 COVID survivors 1 year after hospital discharge, reports new-onset diabetes at 1% of them. Moreover, from the 109 people with diabetes alive after one year (from a total of 189 at discharge), 10% reported impaired glycemic control, 9,2% had to intensify oral therapy, 4,6% required new prescription of insulin (155).

## Discussion

The contribution of diabetes -as a risk factor- in the pathophysiology of COVID-19, 2 years after the pandemic outbreak, is undoubtable. Among diabetes chronic complications and comorbidities such as aging, and obesity, new evidence has put the endothelium in the spotlight. Chronic endothelial dysfunction -seen in DM- predisposes to severe COVID-19 infection by inducing alterations to the glycocalyx and endothelial cells. This leads to increases in leucocyte adhesion, which in turn promotes a procoagulant and antifibrinolytic state. Chronic endothelial dysfunction due to DM together with the direct damage of endothelial cells by SARS-CoV-2 result in further impairment of the microcirculation contributing to the pathogenesis of acute respiratory syndrome and multi-organ failure.

But the cycle is vicious. COVID-19 can cause severe derangements in glucose homeostasis. Glucotoxicity with its consequences, in conjunction with the inflammatory cytokine storm of COVID-19, express increased oxidative stress. Oxidative stress deranges the immune system further and damages the endothelial cells, resulting to metabolic complications -such as increased risk of thromboembolism and multiorgan damage- in individuals with DM (64–66).

The effects of Covid-19 infection on glucose homeostasis are multifactorial, enabling both insulin resistance and β-cell dysfunction. Uncommon requirements of insulin to maintain glycemic control have been observed in all studies, along with extreme fluctuations of glucose. This glucose storm has imposed an emerging interest in research on β-cell invasion, and more specifically in the ability of SARS-CoV-2 to invade and destroy islets through its identified receptors. Besides ACE2, various other receptors have emerged as alternative means of SARS-CoV-2 to enter into cells, that remain to be characterized and further investigated. However, even if SARS-CoV-2 enters β-cells conditionally, it can indirectly affect their function. This is where the endothelium plays an important role because it may connect all COVID-19 target organs, and could explain the β-cell dysfunction, as pericytes and microvasculature are prerequisites for intact β-cell function.

The wealth of existing knowledge about these mechanisms has prompted the scientific community to look into the effect of several antidiabetic medications to COVID-19 outcomes. As expected, DPP4i are of particular and promising interest, not only due to their safety profile in hospitalized patients but also due to the mechanistic knowledge that involves DPP4 as an alternative entry receptor for SARS-CoV-2. Newer agents -namely SGLT-2is and GLP-1 RA- may contribute with their antiinflammatory properties. However, to date, no robust evidence exists to inform practice.

Given the fact that COVID-19 came into being quite recently, it is still unclear whether the dysregulation of glucose metabolism during severe COVID-19 is permanent after recovery or whether SARS-CoV-2 can induce T1DM or T2DM or even a new type of diabetes. Long-term follow up studies are needed to evaluate whether the virus has a diabetogenic impact on individuals with a higher risk for DM or whether it can stimulate a new type of DM.

## Author Contributions

PK wrote the manuscript. SA, AM, and VL edited the manuscript. VL, II, IK, and AM generated the hypothesis and guided the literature review, based on their clinical and research experience in COVID-19 wards and ICU. All authors reviewed the literature, discussed the findings, and reviewed the manuscript. All authors contributed to the article and approved the submitted version.

## Conflict of Interest

The authors declare that the research was conducted in the absence of any commercial or financial relationships that could be construed as a potential conflict of interest.

## Publisher’s Note

All claims expressed in this article are solely those of the authors and do not necessarily represent those of their affiliated organizations, or those of the publisher, the editors and the reviewers. Any product that may be evaluated in this article, or claim that may be made by its manufacturer, is not guaranteed or endorsed by the publisher.
